# Supplementary material for: Human Milk Exosomal MicroRNA: Associations with Maternal Overweight/Obesity and Infant Body Composition at 1 Month of Life
Source: Nutrients. 2021 Mar 27;13(4):1091. doi: 10.3390/nu13041091 (PMC8066780; doi:10.3390/nu13041091)
Supplement: Supplementary file 1 [file nutrients-13-01091-s001.pdf]

Supplemental Table S1. miRNA sequences

miRNA	Taqman Assay ID	Mature Sequence
hsa-miR-29a-3p	002112	UAGCACCAUCUGAAAUCGGUUA
hsa-miR-29b-3p	000413	UAGCACCAUUUGAAAUCAGUGUU
hsa-miR-30b-5p	000602	UGUAAACAUCCUACACUCAGCU
hsa-miR-148a-3p	000470	UCAGUGCACUACAGAACUUUGU
hsa-let-7a-5p	000377	UGAGGUAGUAGGUUGUAUAGUU
hsa-32-3p	002111	CAAUUUAGUGUGUGUGAUAUUU
